# Effectiveness and safety of adjunctive traditional Chinese medicine therapy for constipation after cancer chemotherapy: A protocol for systematic review and meta-analysis

**DOI:** 10.1097/MD.0000000000021770

**Published:** 2020-08-21

**Authors:** Qianxiang Dai, Hang Yan, Xiaoping Wu, Yuan Liu, Fei Huang, Xing Dong

**Affiliations:** a Basic Medical College; b Clinical Medical College, Chengdu University of Traditional Chinese Medicine, Chengdu; c Graduate School of China Academy of Chinese Medical Sciences, Dongzhimen, Dongcheng District, Beijing, China.

**Keywords:** constipation after cancer chemotherapy, meta-analysis, protocol, systematic review, traditional Chinese medicine

## Abstract

**Background:**

As an alternative for constipation after cancer chemotherapy, Chinese medicine has gradually attracted the attention of clinicians based on the theory of syndrome differentiation and treatment. However, due to the lack of evidence-based medical evidence, the author designed the program to evaluate the effectiveness and safety of Chinese medicine.

**Methods:**

From the beginning to August 2020, 8 electronic databases will be searched. Two of our researchers will independently conduct research selection, data extraction, and risk assessment of bias. We will use Review Manager 5.3 software for meta-analysis and heterogeneity assessment. In addition, we will use the grading of recommendations assessment, development, and evaluation to evaluate the evidence quality.

**Results:**

This study will demonstrate an evidence-based review of traditional Chinese medicine (TCM) for constipation after cancer chemotherapy.

**Conclusion:**

The study will provide clear evidence to assess the effectiveness and side effects of TCM for constipation after cancer chemotherapy.

**Trial registration number:**

INPLASY202070027

## 1 Introduction

Cancer is the leading cause of death worldwide,^[1,2]^ with approximately 14.1 million new cancer cases and 8.2 million cancer deaths in 2012 alone.^[3]^ Although advances in modern medicine have improved scanning and cancer detection technologies, the global health burden of cancer is expected to increase in decades, especially in low- and middle-income families and economically developed countries.^[3,4]^ The aging and growth of the population, and the choice of high-risk lifestyles. Exercise, lack of exercise, and Westernization of the diet are considered to be the main factors leading to an increase in global cancer incidence.^[1]^ It is estimated that by 2025, >20 million people will have cancer.^[3]^

Most cancer patients receive treatment or interventional treatment with palliative chemotherapy.^[5–9]^ Although chemotherapy has greatly improved the survival rate of many types of cancer, the cytotoxic side effects are significant and have greatly hindered the application of other beneficial therapies.^[10,11]^ Gastrointestinal side effects such as nausea, vomiting, ulcers, bloating, and constipation, especially diarrhea, are the main obstacles that lead to delays, adjustments, and interruptions of treatment, while greatly affecting the quality of life of many cancer patients.^[5,12]^ Although certain chemotherapeutic drugs may be associated with a higher incidence of chemotherapeutic effects, it is reported that up to 40% of patients receive standard drug therapy and 100% of patients receive super drug therapy.^[13]^ In addition, the incidence of chronic constipation and diarrhea in cancer survivors after treatment is estimated to be as high as 49%, and the episode lasts for >10 years.^[14]^

Constipation is a common and underestimated complication in patients with advanced cancer.^[15]^ Constipation is a subjective and objective feeling, and it is difficult to accept a general definition, although it is widely regarded as a clinical symptom, that is, the frequency of bowl movement decreases and the consistency increases.^[16]^ The constipation rate of patients with advanced cancer is 50% to 87%.^[17]^ Constipation is the third most common symptom. The overall prevalence of patients receiving cytotoxic chemotherapy is 16%, of which 5% is severe and 11% is moderate.^[17,18]^ The basic mechanism of constipation after cancer chemotherapy is roughly defined in the smallest clinical studies available. It is distinguished from secondary constipation, medical treatment, chemotherapy, cancer-induced symptoms (such as anti-nausea and vomiting, and opioid therapy for pain), mainly in the study of disorders.^[19]^ Whereas literature-related scarcity of constipation after cancer chemotherapy it is hard estimates the accurate incidence and severity of radiotherapy in all cancer patients, but specific chemotherapy drugs such as thalidomide, cisplatin, and vinca alkaloids such as vincristine, vinblastine, Vinorelbine promotes true constipation after cancer chemotherapy in up 80 in 90% of patients.^[19,20]^ It was not considered clinically important until constipation caused physical risks or life difficulties. Constipation can cause many obvious symptoms. Patients with severe constipation are often accompanied by severe abdominal distension and severe abdominal pain with dislocation. In addition, rectal tears, hemorrhoids, and rectal fissures leading to bypass and dry urethritis are common complications of constipation. Untreated constipation may be progressive constipation, severe persistent constipation, which may have dangerous complications related to fecal impaction and intestinal obstruction. Fecal impaction, stool failure, and increased pressure in the intestinal cavity can lead to ischemic necrosis of the fascia, pain, bleeding, and perforation. Fecal impaction is also recognized as a cause of urinary incontinence in the elderly. Constipation can also cause fusion, increase peritoneal or pain, cause rapid vomiting with or without vomiting without obvious obstacles, and lead to insufficient absorption of drugs, which seriously affects the tolerance of chemotherapy drugs. There is increasing evidence that self-reported constipation and functional constipation can seriously damage the quality of life, suggesting that this is a serious condition for people who suffer, however, little work has been undertaken to elucidate prevalence and mechanisms.

The management of constipation can be divided into general intervention measures and treatment measures. General interventions include increased physical exercise, fluid intake and fiber consumption, comfort during defecation, privacy and convenience, and elimination of medical factors that may contribute to constipation.^[15]^ Therapeutic interventions for constipation, including constipation after cancer chemotherapy involving the management of rhubarb/rectal mass formation, emollients, osmotic/saline, irritant, and laxatives.^[16]^ Laxatives may be divided into bulk laxatives, osmotic laxatives, emollient laxatives, stimulant laxatives, lubricant laxatives, rectal laxatives according to their mechanism of action.

In recent years, clinical research on the prevention and treatment of chemotherapy-related constipation by traditional Chinese medicine (TCM) has increased day by day, and its curative effect is remarkable.^[21–29]^ In China and some Southeast Asian countries, Chinese medicine has long been used to treat constipation. However, whether TCM treatment of constipation after cancer chemotherapy is also safe and effective is still controversial. Owing to the lack of evidence-based medical evidence, we designed this protocol to evaluate TCM's effectiveness and safety.

## 2 Methods

### 2.1 Protocol register

Our systematic review and meta-analysis protocol have been registered on the INPLASY international prospective register of systematic reviews (INPLASY202070027). We prepared the plan according to the preferred report project of systematic review and meta-analysis protocol guide.^[30]^ The final report will follow PRISMA's recommendation to systematically review the report to incorporate the report into the network meta-analysis of healthcare interventions.^[31]^

### 2.2 Ethics

Since the program does not require patient recruitment or collection of personal information, no further ethical approval is required.

### 2.3 Database search strategy

Use computer search and manual search for all published articles. The searched databases include PubMed, EMBASE database, Cochrane central controlled trial registration database, Chinese biomedical database, Chinese national knowledge infrastructure, Chinese scientific journal database, and Wanfang database. All randomized controlled trials (RCT) of traditional Chinese medicine used to treat constipation after chemotherapy will be searched until December 2021. The specific search strategy will be formulated with a specific database. Among them, the author lists the search strategy of the PubMed database (Table 1), and will be supplemented by manually searching for relevant literature.

**Table 1:**
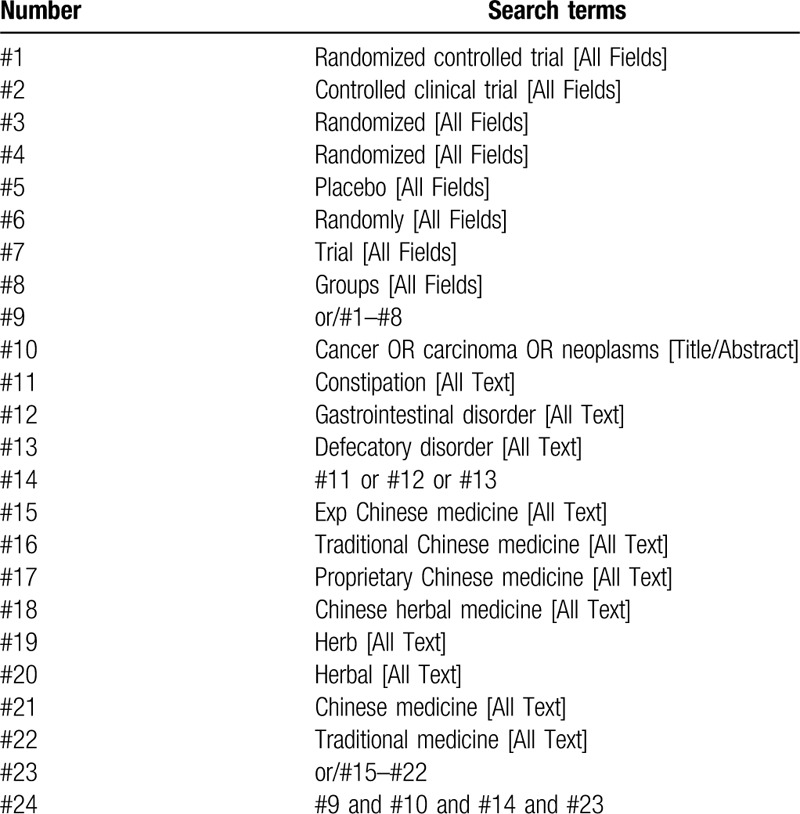
Search strategy used in the PubMed database.

At the same time, we also plan to manually search the published references of relevant systematic reviews and reviews. There is no date limit, country, publication status, or publication year limit.

### 2.4 Eligibility criteria and elimination criteria

#### 2.4.1 Types of participants

According to the National Comprehensive Cancer Network (NCCN) Clinical Guidelines for Cancer, cancer patients confirmed by pathology, cytology or imaging, and patients undergo chemotherapy treatment and cause constipation. The diagnosis of constipation complies with Roman standards.

#### 2.4.2 Types of interventions

Observation group: Traditional Chinese medicine is used alone or in combination with other treatment methods. The types of Chinese medicines and methods of combination will be ignored.

Control group: Other treatments (including any other non-Chinese medicine treatment) or combined with fake Chinese medicine.

#### 2.4.3 Types of outcome measures

Main outcomes: The scoring standard of constipation efficacy refers to “Roman Standard,” abdominal distension: 0 points for no abdominal distension, 1 point for mild abdominal distension, 2 points for more obvious abdominal distension, 3 points for obvious abdominal distension and affecting daily life. Fecal traits: According to the “Bristol stool profile,” type 1 is a separate hard block; type 2 is a clump; type 3 is a dry and cracked sausage; type 4 is a soft sausage; type 5 is a soft clump; 6 Type 7 is muddy; Type 7 is watery stool; Types 4 to 7 count 0 points; Type 3 counts 1 point; Type 2 counts 2 points; Type 1 counts 3 points. Defecation time: <10 minutes counts 0 points, 10 to 20 minutes counts 1 point, 20 to 30 minutes counts 2 points, >30 minutes counts 3 points. Defecation effort: 0 points for smooth defecation, 1 point for difficulty in defecation, 2 points for difficulty in defecation, 2 points for difficulty in defecation, 3 points for defecation or enema.

Additional outcomes: Quality of life, improvement of clinical symptoms, such as fatigue, and loss of appetite, adverse events (AEs).

#### 2.4.4 Type of study

Only randomized controlled trials (RCT) meet our requirements.

### 2.5 Study selection and data collection

**Figure 1:**
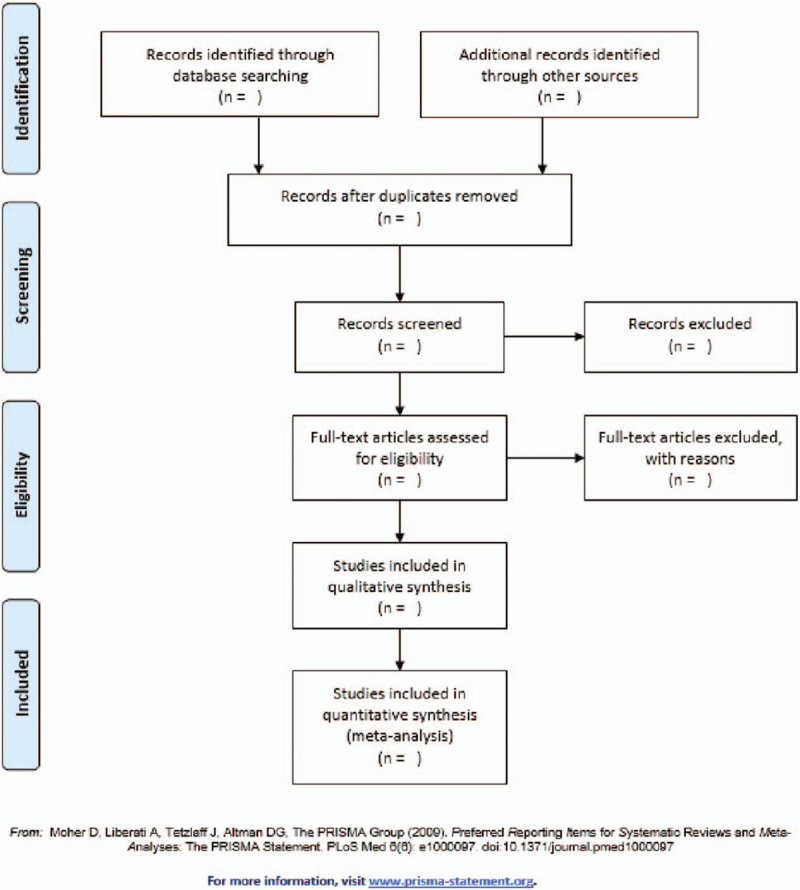
Study flow diagram, illustrate the process of studies selection.

We will use Literature management software for document management. First, we will use the software to classify and organize documents, and remove duplicate documents based on the title and abstract. Second, the 2 researchers will independently screen relevant studies that meet the inclusion criteria based on the article's title, abstract, and keywords. Then, for uncertain research, we will download the full text for evaluation. The process will be completed independently by 2 researchers, and then the results will be cross-checked. If the conclusions of the 2 evaluators are inconsistent, the differences can be resolved through discussion. In the list of excluded studies, we will report the reasons for the excluded studies in the full text review. The process of research screening is shown in Fig. 1.

Next, the 2 researchers will use pre-designed Microsoft Excel data extraction tables to independently extract the data in the study. The data items we plan to extract include:

1. Research characteristics (author, journal, year of publication, randomization method, blind method, etc).
2. Participants (sample size, age, disease duration, disease diagnosis criteria, etc).
3. Intervention (name of traditional Chinese medicine, type of treatment, dosage form, clinical dose, treatment process, etc).
4. Control (treatment type, treatment process, dosage form, clinical dose, etc).
5. Results (outcome, type of result measure, adverse event, etc).

The 2 researchers will extract data independently. If you have any objections, you can discuss or consult with the third author. If there are missing data in the study, we will first contact the corresponding author to obtain the data.

### 2.6 Dealing with missing data

We will get the missing data by contacting the study authors, and discuss the reasons, degree, nature, and how to deal with the missing data in each study. If data are still not available, we will only conduct a descriptive review of the study.

### 2.7 Literature quality assessment

The 2 researchers will independently conduct a bias risk assessment of studies that meet the inclusion criteria based on the Cochrane bias risk tool. It mainly includes 7 aspects: generation of random sequence, concealment of allocation, blindness of participants and personnel, blindness of result data, incompleteness of result data, selective reports, and other biases. If there are differences during the evaluation process, they will be resolved through discussion.

### 2.8 Statistical analysis methods

If no fewer than 2 studies meet the inclusion criteria, we will conduct a paired meta-analysis. Odds ratio will be used to assess the magnitude of the impact of dichotomous variables, while the magnitude of the impact of continuous variables will be assessed using the mean difference. Since the included studies may lead to heterogeneity in methods, clinical and statistical analysis, we will use a random effects model to synthesize the data.^[32]^ Heterogeneity is inevitable due to the methods and clinical diversity that always exist in meta-analysis. We will evaluate the heterogeneity of the study by calculating *I*^2^. The interpretation of *I*^2^ will be based on the threshold level proposed in the Cochrane collaboration. If there is significant heterogeneity affecting the results, we will conduct subgroup analysis and meta-regression analysis to study the potential influencing factors, such as the participant's age, sample size, disease duration, treatment process, and study quality. Sensitivity analysis will be used to check the stability of the results. If the number of studies is >10, we will also assess the publication bias of the included studies. As we all know, TCM research has always involved syndrome differentiation.^[33]^ However, this study only evaluates whether Chinese medicine is effective and safe for constipation after chemotherapy. Therefore, this study did not separately conduct a meta-analysis of different TCM syndromes. In order to explore whether TCM syndromes cause heterogeneity, we will conduct a subgroup analysis of TCM syndrome types.

### 2.9 Grading of recommendations assessment, development, and evaluation quality assessment

We will use grading of recommendations assessment, development, and evaluation (GRADE) to assess the quality of evidence and the strength of the main result recommendation.^[34,35]^ Five factors can reduce the quality of evidence: study limitations (risk of bias), inconsistency, discontinuity, publication bias, and inaccuracy. And 3 factors can improve the quality of evidence: residual confusion, dose–response gradient, and large effects. The quality of evidence will be divided into 4 levels: extremely low, low, medium, and high. This step will be performed using GRADE software.

## Author contributions

**Conceptualization:** Qianxiang Dai, Hang Yan, Yuan Liu.

**Data curation:** Qianxiang Dai.

**Formal analysis:** Hang Yan, Xiaoping Wu.

**Methodology:** Fei Huang, Xing Dong.

**Project administration:** Qianxiang Dai.

**Supervision:** Xing Dong.

**Validation:** Hang Yan.

**Writing – original draft:** Qianxiang Dai, Yuan Liu.
